# Supplementary material for: A First-Principles Study on the Multilayer Graphene Nanosheets Anode Performance for Boron-Ion Battery
Source: Nanomaterials (Basel). 2022 Apr 9;12(8):1280. doi: 10.3390/nano12081280 (PMC9030437; doi:10.3390/nano12081280)
Supplement: Supplementary file 1 [file nanomaterials-12-01280-s001.zip › nanomaterials-1651607-supplementary.pdf]

nanomaterials

Supplementary Information

A First-Principles Study on the Multilayer Graphene Nanosheets Anode Performance for Boron-Ion Battery

Mustapha Umar [1], Chidera C. Nnadiekwe [1], Muhammad Haroon [1], Ismail Abdulazeez [2,*], Khalid Alhooshani [1,3], Abdulaziz A. Al-Saadi [1,3] and Qing Peng [4,5,6,*]

[1] Chemistry Department, King Fahd University of Petroleum and Minerals, Dhahran 31261, Saudi Arabia; g201708770@kfupm.edu.sa (M.U.); g201707330@kfupm.edu.sa (C.C.N.); muhammad.hanif@kfupm.edu.sa (M.H.); hooshani@kfupm.edu.sa (K.A.); asaadi@kfupm.edu.sa (A.A.A.-S.)
[2] Interdisciplinary Research Center for Membranes and Water Security, King Fahd University of Petroleum and Minerals, Dhahran 31261, Saudi Arabia
[3] Interdisciplinary Research Center for Refining and Advanced Chemicals, King Fahd University of Petroleum and Minerals, Dhahran 31261, Saudi Arabia
[4] Interdisciplinary Research Center for Hydrogen and Energy Storage, King Fahd University of Petroleum and Minerals, Dhahran 31261, Saudi Arabia
[5] Physics Department, King Fahd University of Petroleum and Minerals, Dhahran 31261, Saudi Arabia
[6] KACARE Energy Research and Innovation Center at Dhahran, Dhahran 31261, Saudi Arabia
* Correspondence: ismail.abdulazeez@kfupm.edu.sa (I.A.); qing.peng@kfupm.edu.sa (Q.P.)

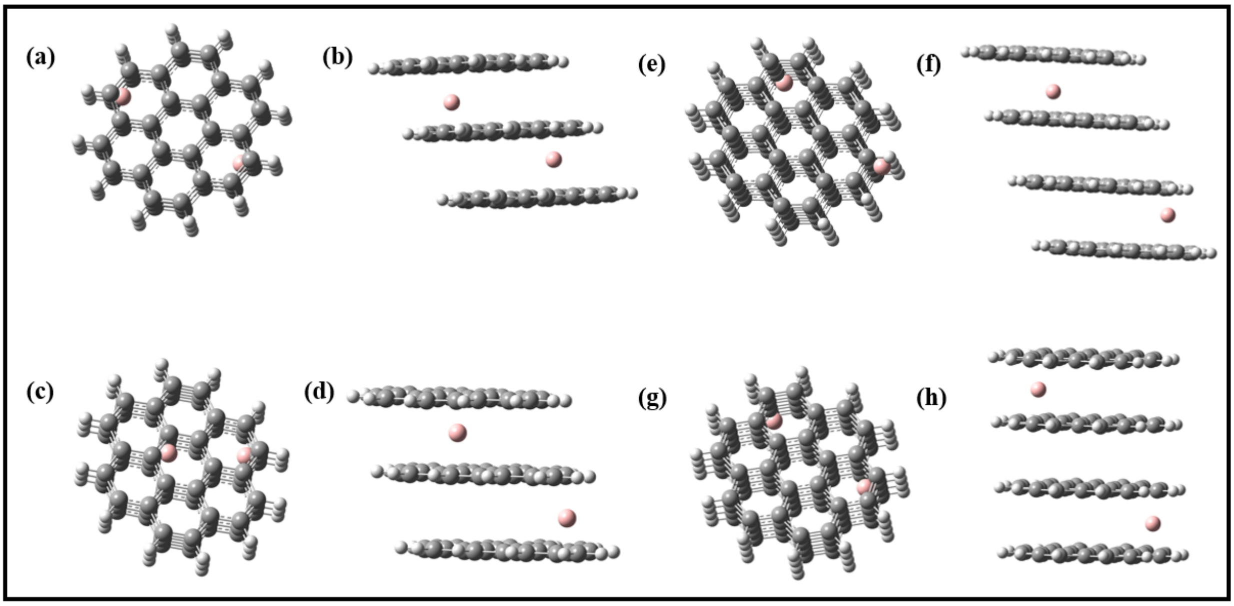

Figure S1. Optimized structures of **(a)** and **(b)** 2B@TG (side and topviews), **(c)** and **(d)** 2B@TG (side and topviews), **(e)** and **(f)** $2B^{3+}$@TTG (side and topviews), **(g)** and **(h)** $2B^{3+}$@TTG (side and topviews).

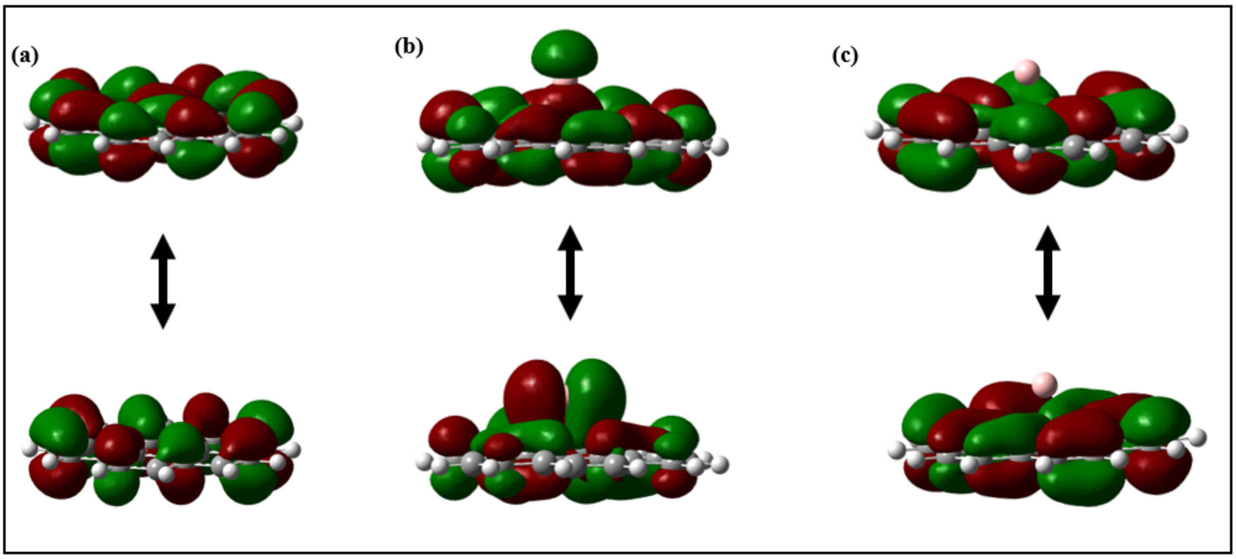

Figure S2. Molecular Orbital HOMO (Lower) and LUMO (upper) of (a) Monolayer Graphene Sheet (MG), (b) B@MG, (c) B^{3+}@MG.

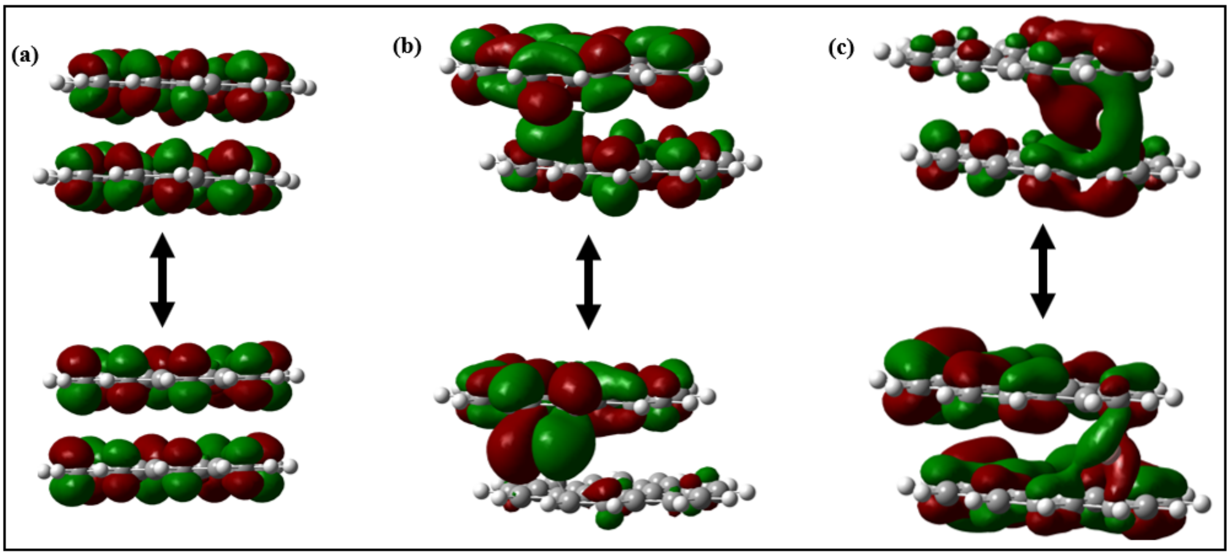

Figure S3. Molecular Orbital HOMO (Lower) and LUMO (upper) of (a) Bilayer Graphene Sheet (BG) (b) B@BG, (c) B^{3+}@BG.

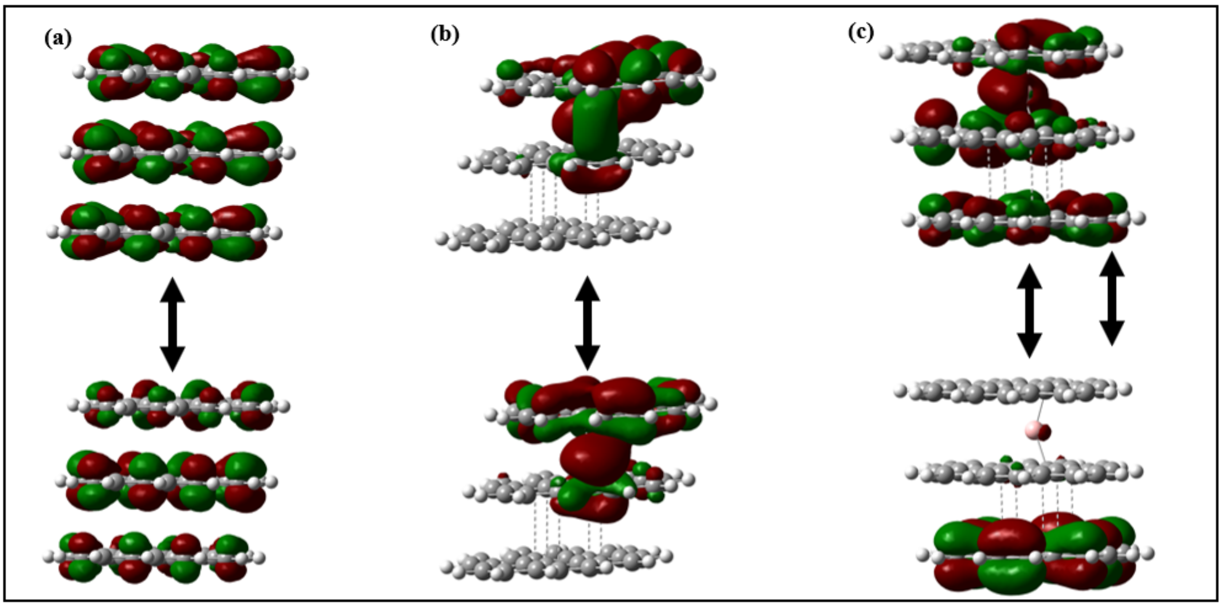

Figure S4. Molecular Orbital HOMO (Lower) and LUMO (upper) of (a) Trilayer Graphene Sheet (TG), (b) B@TG, (c) B^{3+}@TG.

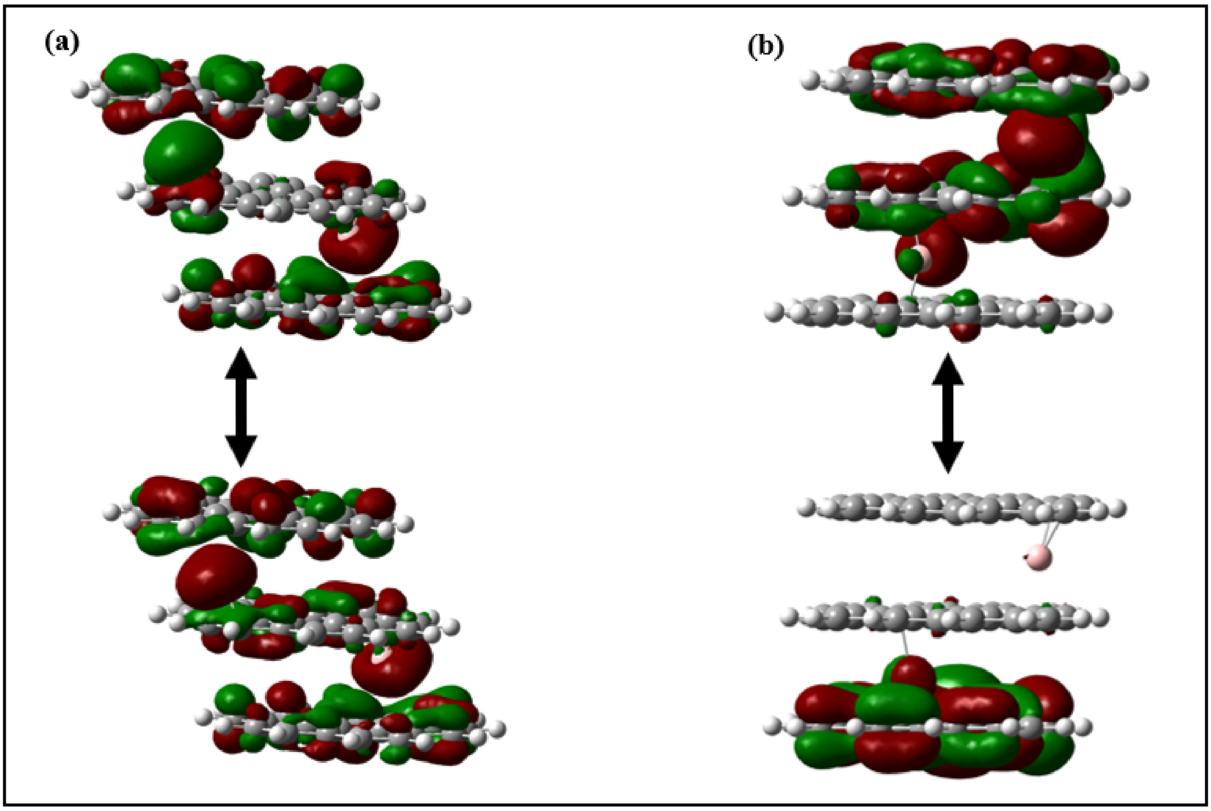

Figure S5. Molecular Orbital HOMO (Lower) and LUMO (upper) of (a) 2B@TG and (b) $2B^{3+}$@TG.

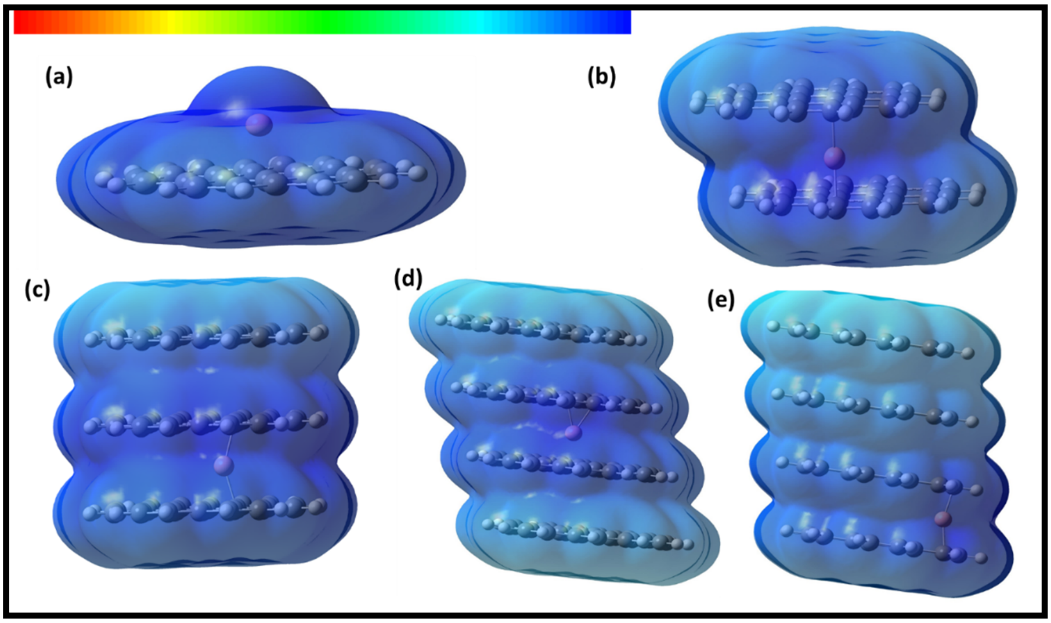

Figure S6. Electrostatic potential (ESP) map of (a) B^{3+}@MG, (b) B^{3+}@BG, (c) B^{3+}@TG, (d) B^{3+}@TTG_sym and (d) B^{3+}@TTG_asym.

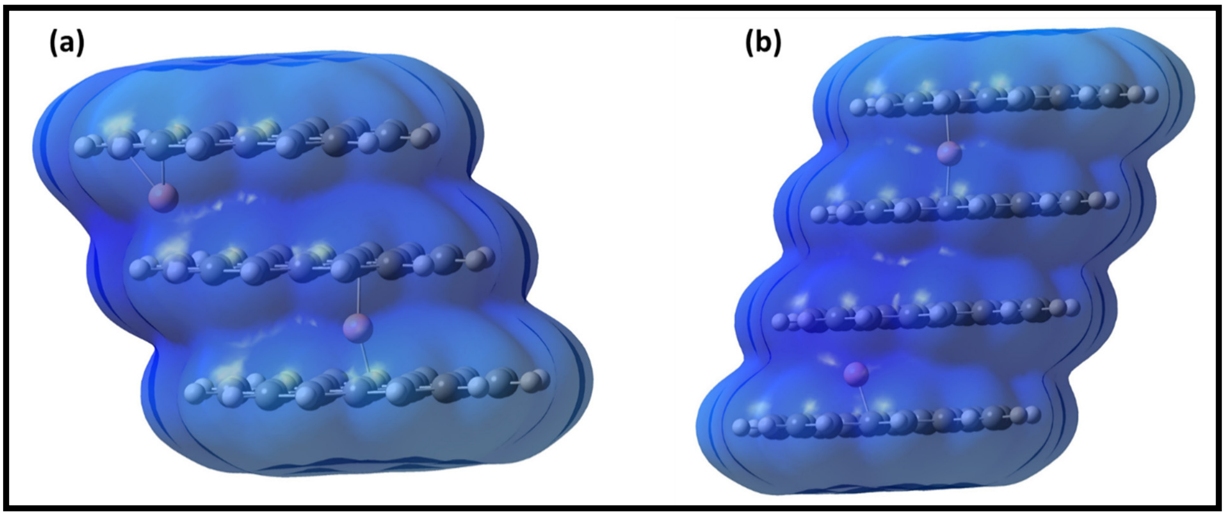

Figure S7. Electrostatic potential (ESP) (a) $2B^{3+}$@TG and (b) $2B^{3+}$@TTG.

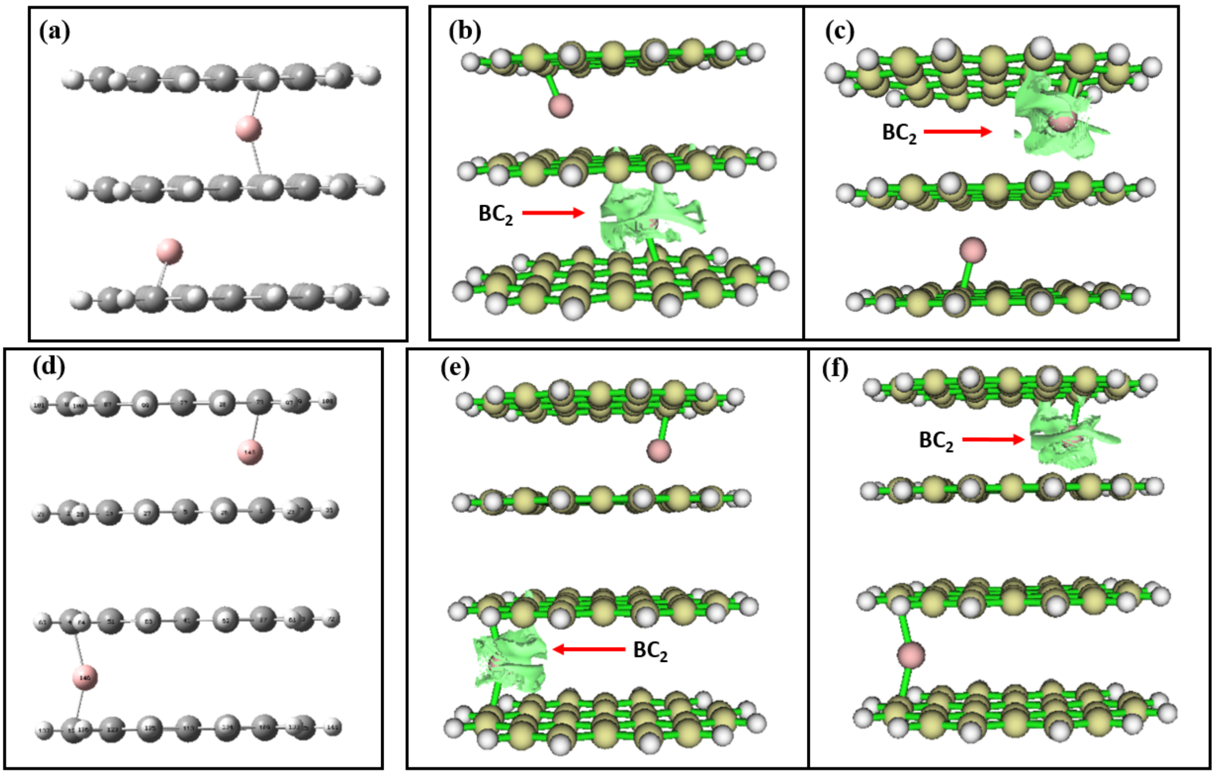

Figure S8. The reduced density gradient (RDG) isosurfaces analyses of (**a**), **(b)** and **(c)** 2B^{3+}@TG (complex and RDG), and **(d)**, **(e)** and **(f)** 2B^{3+}@TTG (complex and RDG). Where blue regions correspond to strong hydrogen bonds; red regions indicate strong steric effects, whereas green regions describe strong van der Waals interactions.

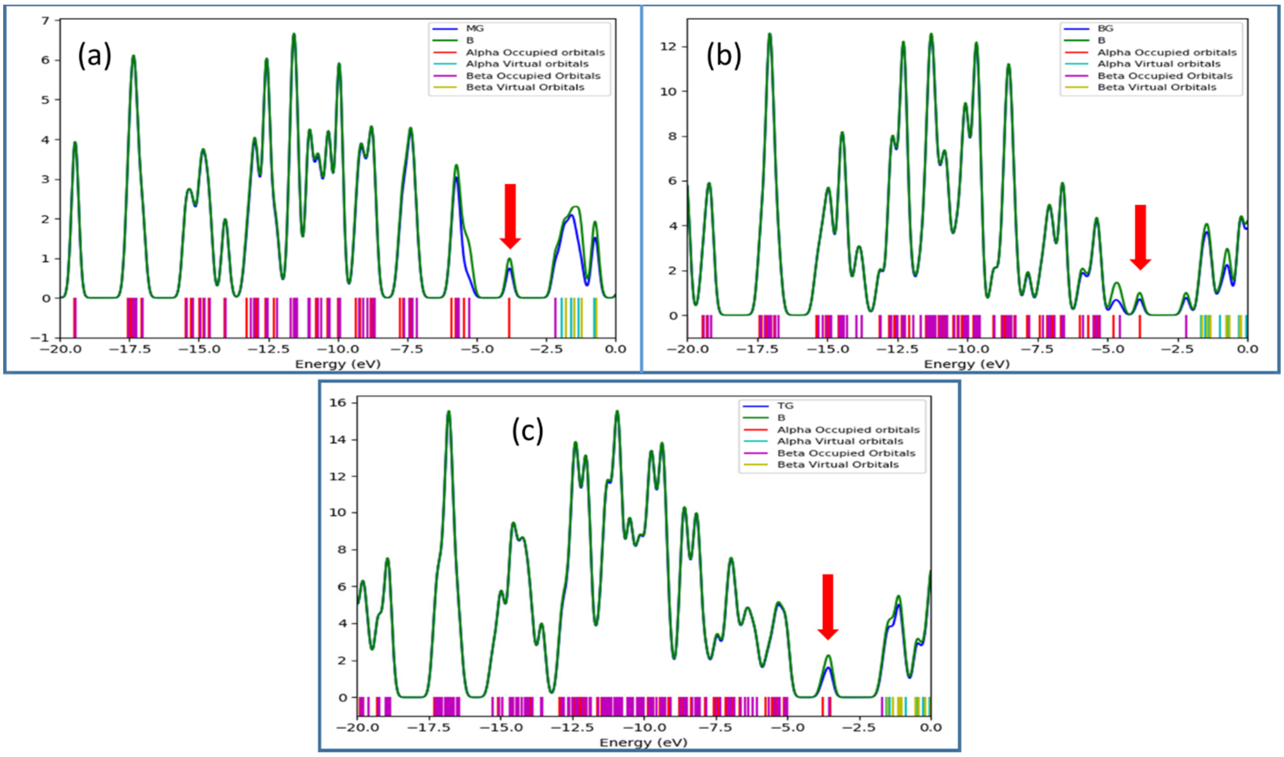

Figure S9. Partial density of states (PDOS) plots (a) B@MG (b) B@BG and (c) B@TG.

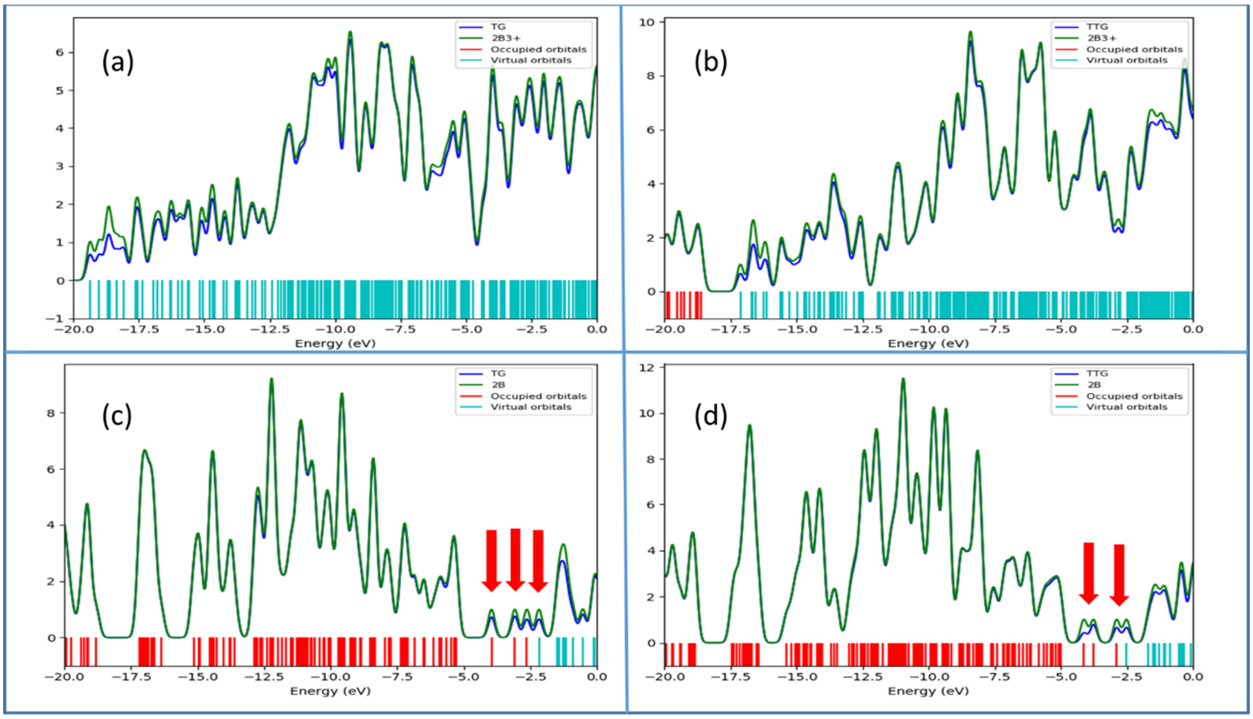

Figure S10. Partial density of states (PDOS) plots (a) $2B^{3+}$@TG (b) $2B^{3+}$@TTG (c) B@TG_sym and (d) 2B@TTG.

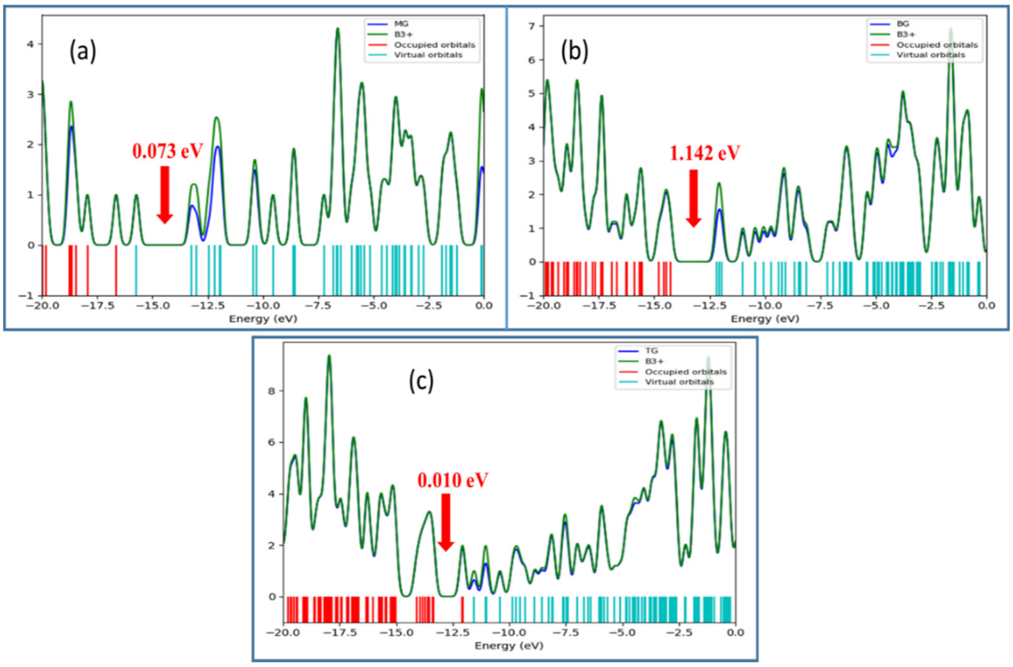

Figure S11. Partial density of states (PDOS) plots of (a) B^{3+}@MG (b) B^{3+}@BG and (c) B^{3+}@TG.

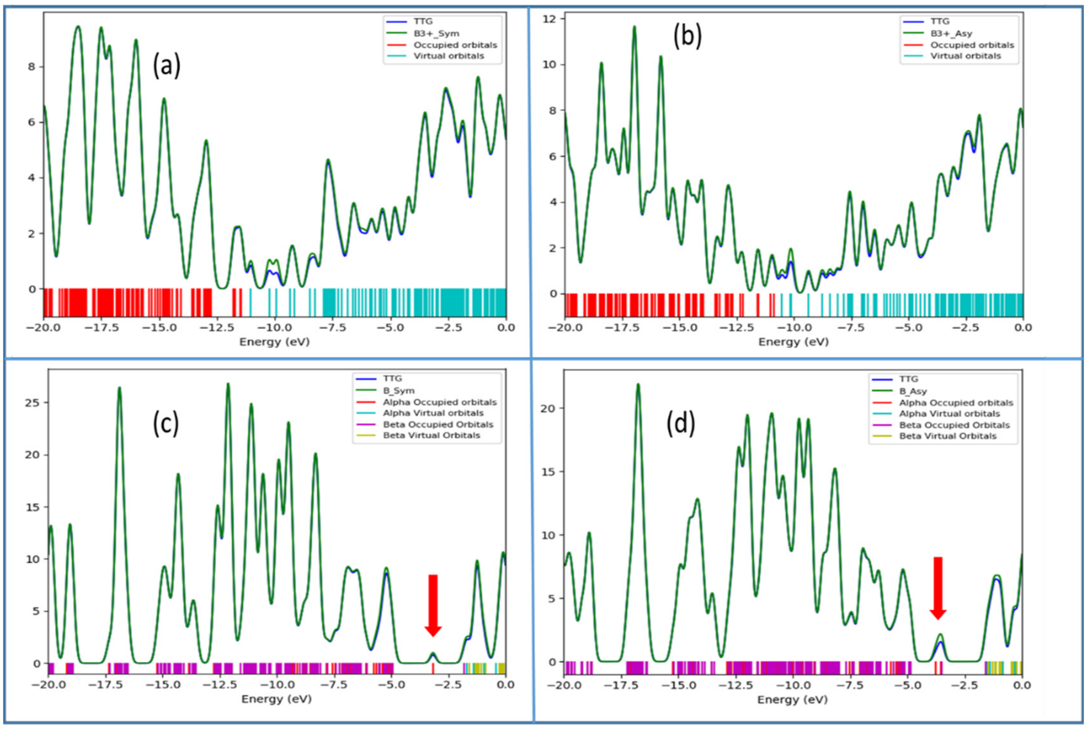

Figure S12. Partial density of states (PDOS) plots of (a) B^{3+}@TTG_sym (b) B^{3+}@TTG_asym (c) B@TTG_sym and (d) B@TTG_asym.

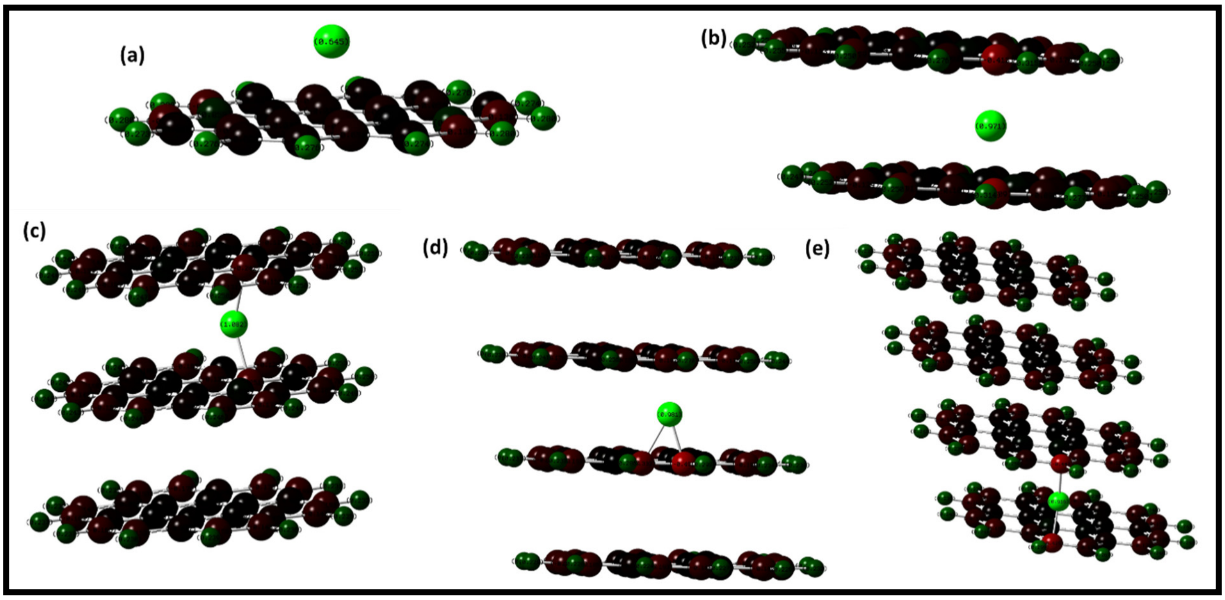

Figure S13. Natural bond orbital charges of (a) B^{3+}@MG, (b) B^{3+}@BG and (c) B^{3+}@TG, (d) B^{3+}@TTG_sym and (e) B^{3+}@TTG_asym.

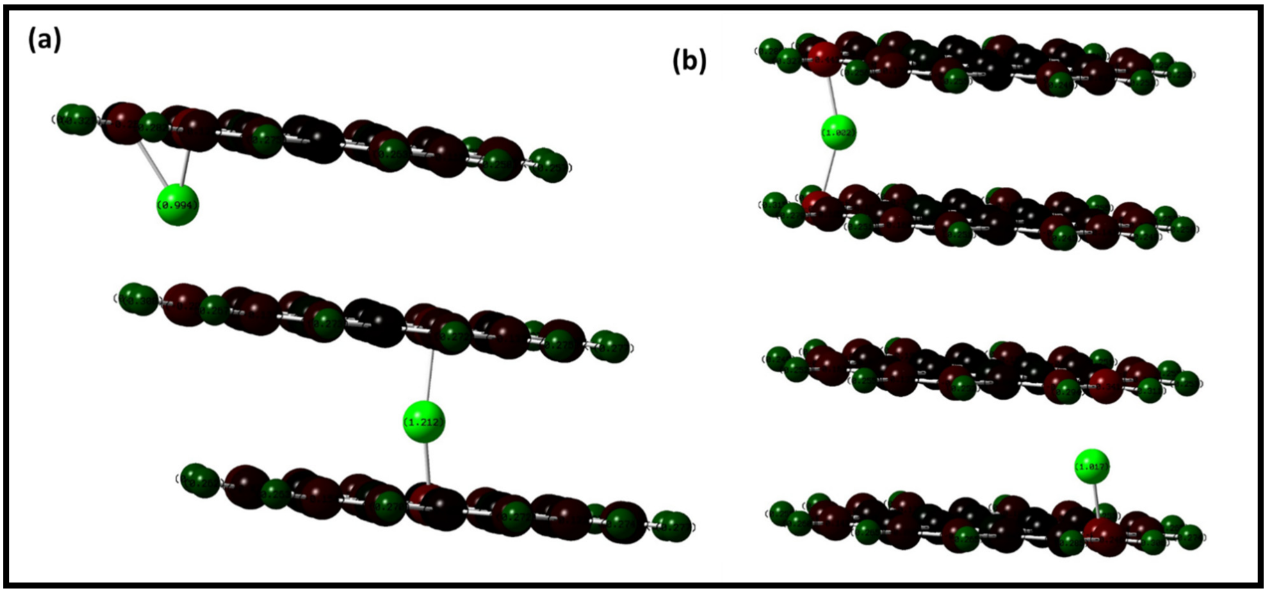

Figure S14. Natural bond orbital charges of (a) $2B^{3+}$@TG and (b) $2B^{3+}$@TTG.

Table S1. Electronic properties.

Complexes	E_{HOMO} (eV)	E_{LUMO} (eV)	E_g (eV)	μ (eV)	η (eV)	χ(eV)	E_a (eV)
**MG**	−5.085	−2.242	2.843	13.42	1.421	3.663	2.242
**BG**	−4.883	−2.249	2.635	12.72	1.317	3.566	2.249
**TG**	−4.760	−2.233	2.527	12.23	1.264	3.496	2.233
**FGS**	−4.622	−2.151	2.472	11.47	1.236	3.386	2.151
**B@MG**	−4.857	−3.125	1.731	15.93	0.866	3.991	3.125
**B^{3+}@MG**	−16.31	−16.23	0.073	264.7	0.036	16.27	16.23
**B@BG**	−4.240	−3.122	1.118	13.55	0.559	3.681	3.122
**B^{3+}@BG**	−13.98	−12.84	1.142	179.8	0.571	13.41	12.84
**B@TG**	−3.407	−2.614	0.793	9.061	0.396	3.010	2.614
**B^{3+}@TG**	−11.96	−11.97	0.010	143.1	−0.005	11.96	11.97
**B@TTG_asym**	−3.347	−2.507	0.840	8.567	0.420	2.927	2.507
**B^{3+}@TTG_asym**	−10.85	−10.84	0.010	117.5	0.005	10.84	10.84
**B@TTG_sym**	−4.688	−2.680	2.009	13.57	1.004	3.684	2.680
**B^{3+}@TTG_sym**	−11.28	−11.28	0.003	127.1	0.002	11.28	11.28
**2B@TG**	−2.862	−2.787	0.075	7.979	0.038	2.825	2.787
**$2B^{3+}$@TG**	−20.08	−19.84	0.241	398.4	0.121	19.96	19.84
**2B@TTG**	−3.099	−3.059	0.040	9.479	0.020	3.079	3.059
**$2B^{3+}$@TTG**	−18.33	−17.66	0.672	323.9	0.336	18.0	17.66
